# Why a registry of Chronic Urticaria (CUR) is needed

**DOI:** 10.1186/s40413-017-0147-2

**Published:** 2017-05-16

**Authors:** R. M. Gómez, E. Jares, G. W. Canonica, I. Baiardini, G. Passalacqua, M. Sánchez Borges, A. P. Kaplan, Carlos E. Baena-Cagnani

**Affiliations:** 1 Fundación Ayre at Instituto Médico Alas, Sarmiento 771, 330-31, 4400 Salta, Argentina; 2 Allergy & Asthma Unit, Hospital San Bernardo, Salta, Argentina; 3 Libra Foundation, Buenos Aires, Argentina; 4 Allergy & Respiratory Diseases Clinic, University of Genova, IRCCS AOU S.Martino, Genoa, Italy; 5 Allergy & Immunology Dpt, Centro Médico Docente La Trinidad, Caracas, Venezuela; 6 The Medical University of South Carolina, Charleston, SC USA

**Keywords:** Chronic Urticaria, Registry, Tools, Approach, Management

## Abstract

Chronic urticaria (CU) has a major effect on patients’ quality of life. While there have been progressive advances regarding its pathogenesis and treatment, much remains to be done.

Registries of other chronic non-communicable diseases have shown many benefits, such as additional basic knowledge and management approaches to diabetes mellitus. Standards of care as well as diagnostic approaches can be elaborated and compared from different sites, using validated instruments. Registries in allergic diseases are also becoming well recognized, and the first registry on CU, accessible from SLaai’s webpage, includes parameters for identification, evaluation and management. In our vision, informatics strategies have the potential to improve care for chronic illnesses such as CU. The registry represents a valid instrument from which to obtain a sufficient sample size for epidemiological studies and/or clinical research planning, including feasibility and potential enrollment. It can also provide invaluable data for adapting guidelines to local populations, as well as diagnostic approaches and cost-effective interventions in the context of organizational efforts to improve patient care.

**Electronic supplementary material:**

The online version of this article (doi:10.1186/s40413-017-0147-2) contains supplementary material, which is available to authorized users.

## Why CU represents a major health problem?

Urticaria is characterized by wheals and flares, with or without angioedema, presenting a wide spectrum of severity. Considered a frequent disease, its prevalence has been reported from less than 1% to over 10%, and it is estimated that 20% of the population will suffer an urticarial episode sometime in their life [1, 2]. In children, the prevalence was reported to be about 5% [3].

Urticaria is generally classified by its duration, i.e. acute if lasting < 6 weeks, or chronic when lasting more than 6 weeks. Chronic urticaria involves two primary subgroups according to whether symptoms occur spontaneously (Chronic Spontaneous Urticaria - CSU) or is induced by a demonstrable stimulus (Chronic Inducible Urticaria - CIU), and individual conditions are described according to their underlying etio-pathology or purported mechanism of induction [4]. Considering patients with no identifiable stimulus, about 10% have circulating IgG anti-IgE and 30 to 40% have IgG antibody to the alpha subunit of the IgE receptor. However, more than half of patients lack of these autoantibodies. In either case the disorder is now called chronic spontaneous urticaria [2].

CSU and other chronic forms of urticaria adversely affect performance at work and school, decrease quality of life, and belong then, to the group of severe “allergic” diseases [1, 5].

Second-generation antihistamines are the mainstay of pharmacological treatment in adults and children, aimed at relief of symptoms, with dose adjustment for pediatric use. However, gaining control of symptoms with anti-histamines is achieved in less than half of patients with regular doses, with a limited improvement of this proportion with four fold regular doses [6, 7]; hence a substantial need for new therapeutic strategies emerges. Addition of LTR antagonists or changing anti H1 receptor antagonists plus short cycles of steroids are commonly considered when insufficient control is obtained; but ultimately the treatment involves the use of immune-modulators like cyclosporine, hydroxychloroquine, dapsone and others for those refractory situations [6–9]. Because of the potential side effects of steroids, cyclosporine and dapsone, anti IgE biological therapy (omalizumab) has emerged as an effective and safe alternative. Omalizumab reduces free IgE levels, and decreases responsiveness to stimuli acting through the IgE receptor, reverses blood basopenia, and limits recruitment of basophils into skin lesions [10, 11]. It may emerge as the agent of choice for patients refractory to anti histamines [12].

The lack of control in a remarkable proportion of these chronic patients evidences many unmet needs regarding its patho-physiology and treatment [6, 7].

## Why a registry?

Registries from real life patients and databases constitute key instruments to develop health policies, diagnosis and treatment guidelines, and clinical research in order to provide the best patient care.

They are valuable tools with the potential to transform the way chronic diseases are approached. To date, little work has been done to determine how a chronic disease registry could improve patient management. Some successful initiatives have been reported; diabetes is an example [13].

There is an endless opportunity for the exchange of health information that could be developed. Computerized registries offer the possibility to identify both common characteristics and differences in the magnitude of the illness and its natural history. The severity and quality of life involvement, and the efficacy of recommended treatment and any undesirable side effects can be catalogued. Chronic conditions are ideal subjects to be explored.

Development of registries could potentially help to determine standards of care. In 2007, Young et al. performed a systematic review on the usefulness of having clinical information integrated; the main recommendation was that interactive (disorder-specific) pathways should be implemented to quickly provide clinicians with patient clinical status, treatment history, and decision support [14].

A European initiative to register patients receiving orphan drugs provides needed data on efficacy and safety since marketing authorization is usually obtained with necessary but incomplete evidence. A specific report collected by Orphanet is available on the web [15].

Registries in Allergology provided of valuable information, quite difficult to obtain with other methodologies. A nice example is the online survey on anaphylaxis in Latin America (OLASA), which evidenced that half of patients, did not have access to immediate treatment for anaphylaxis, and also fewer than 40% of patients have received epinephrine, just to mention some of the remarkable information obtained from this registry [16].

Another valuable registry is the Drug Allergy and Hypersensitivity Database, evaluating patients having undergone a standardized procedure on drug intolerance. Information like a low risk when administering cefuroxime in B lactams’ sensitive patients is one example, as well as additional evaluations on side chain cross reactions [17].

## What should a CU registry include?

As urticaria is a heterogeneous group of diseases, important CU features must be considered. The definition of each type of urticaria, as well as tools employed for diagnosis must provide easy access to the investigator.

In typical chronic spontaneous urticaria, wheals arise spontaneously with no identified stimuli. While parasite infection may be a significant association in some countries, the presence of eosinophilia should lead to a stool analysis for ova and parasites, at minimum. Otherwise patients presenting with chronic spontaneous urticaria with a normal physical exam and no other suggestive complaints should not be evaluated for food allergy or occult infections [18].

In order to evaluate disease activity and response to treatment, validated scoring systems like the urticaria activity score should be used [19].

Psychological stress can exacerbate CSU symptoms, even though it is not a cause, and at least in some patients taking this fact into account may be important for the management [20–22]. Health Related Quality of Life (HRQL) is increasingly used in epidemiological studies, with a paramount importance in chronic conditions. Validated CSU specific questionnaires should be implemented and they are available in different languages [23–26]. CSU has a detrimental effect on both objective functioning and subjective well - being comparable to those reported from patients with coronary artery disease, and both health status and subjective satisfaction are lower than in respiratory diseases [27, 28].

Efficacy and side effects of prescribed treatment should also be assessed since there are still many unmet needs [1, 6].

## CU first registry

The Libra and Ayre Foundations from Argentina are devoted to research and education regarding allergic diseases. Their members developed and provide the maintenance of an electronic database registry for CU that was made available to SLaai authorities for its dissemination, as part of the activities offered to their members, in Spanish.

The present initiative was granted from the declared foundations to SLaai, being guided by its Scientific Secretary. Data is owned by SLaai’s Scientific Secretariat, and is entered by any identified allergologist from the region. All allergologists from Latin America willing to participate are invited.

The scientific board is formed by two members from Executive Committee (one is the Scientific Secretary), a member from Urticaria Scientific Committee and four external advisory board persons (authors).

About ethics approval, notwithstanding regulation concerning observational retrospective research exempt from IRB/IEC approval (Eg 10.1093/ije/dyp164), two independent ethics committees were consulted (Medical Council of Salta & Clinical Studies Foundation of Buenos Aires, Argentina). Both did agree on the unnecessary written informed consent, based on the minimum risk of the proposed project. However, one of them suggested informing the patient on the initiative and the anonymous feature of the registry if seen, just like it is stated in the web page.

Two non-governmental non-profit foundations (Ayre & Libra) supported database development and maintenance of it. No funding for authors or participants was provided. There is no role of the industry at all.

The initiative is supported by the Latin American Society of Allergy, Asthma and Immunology, and no other support or endorsement was searched by now.

The present registry (accessible through SLaai’s webpage as Registro Iberoamericano de Urticaria Crónica Espontánea) involves several aspects of CU identification, evaluation and management [29].

Sections were developed considering key elements for electronic surveys, and how the outcomes could be interpreted [30]. Once available, validated instruments like UAS7 and CU-Q2oL were used [19, 26].

It begins with criteria for patient eligibility for the registry. If acceptable, demographic data of patients and the reporting physicians are registered.

The next section deals with CU time elapsed, identification of triggers and severity of symptoms, followed by information regarding quality of life aspects reported by patients.

Then, all laboratory measurements available are tabulated and clinical evaluations performed.

Finally, interventional strategies as well as efficacy and side effects of treatments prescribed are reviewed.

The full content of the registry accessed on line is available as Additional file 1. It is a cross sectional registry, looking for all the information available to assisting physician, and does not intend to follow up treatment efficacy or evolution of the variables reported.

The present registry is completely anonymous for patients and blinded for participants, with just two persons (investigators who developed the database) having access to the collected data. Once the data collection is considered appropriate and closed (estimated in over two hundred reports), principal investigators will maintain a secured copy and the registry will be deleted.

A potential limitation on the present registry is the generalization of these data, obtained from patients assisted by Latin American allergologists. This limitation could also highlight some particular characteristics to be compared with non-Latin American populations, as well as the potential differences between Latin American countries.

Even recognizing that there is no standardized approach for these patients, the present registry does not intend to provide one. Instead, it is devoted to search for peculiarities and common features, with the potential to find pitfalls or unmet needs from current available guidelines about CU diagnostic methods and management in the present population.

By the time this initiative was developed and communicated, it was the first to our knowledge. However, the CURE project available now at http://www.urticaria-registry.com evidenced similarities of variables such as characteristics of patients having CU, causal factors, related triggers, treatment response and some quality of life parameters. The differences compared to the present registry (a cross sectional one from retrospective data) are not searching for duration and course of disease, disease activity, quality of life impairment, absence from school or work and health care costs.

## Conclusions

In our vision, informatics strategies have the potential to improve care for chronic illnesses such as CU. Registries usefulness is well recognized in allergic diseases [31], and the present registry represents a valid instrument from which to obtain a sufficient sample size for epidemiological studies and/or clinical research planning, including feasibility and potential enrollment. It can also provide invaluable data for adapting guidelines to local populations, as well as diagnosis approach and cost-effective interventions in the context of organizational efforts to improve patient care.

## Additional file

Additional file 1: The full content of the registry accessed on line. (DOCX 5526 kb)

## Abbreviations

Anti H1: Anti Histamine 1
CIU: Chronic Inducible Urticaria
CSU: Chronic Spontaneous Urticaria
CU: Chronic Urticaria
CU-Q2oL: Chronic Urticaria – Quality 2 of Life
CUR: Chronic Urticaria Registry
CURE: Chronic Urticaria Registry
HRQL: Health Related Quality of Life
IgG/IgE: Immunoglobulin G/Immunoglobulin E
LTR: Leuco-trienes Receptor
OLASA: On-line Latin American Survey in Anaphylaxis
SLaai: Latin American Society of Allergy, Asthma & Immunology (in Spanish)
UAS7: Urticaria Activity Score in 7 days
