# Supplementary figures and images for: Why a registry of Chronic Urticaria (CUR) is needed
Source: World Allergy Organ J. 2017 May 16;10(1):16. doi: 10.1186/s40413-017-0147-2 (PMC5433157; doi:10.1186/s40413-017-0147-2)

Additional file 1


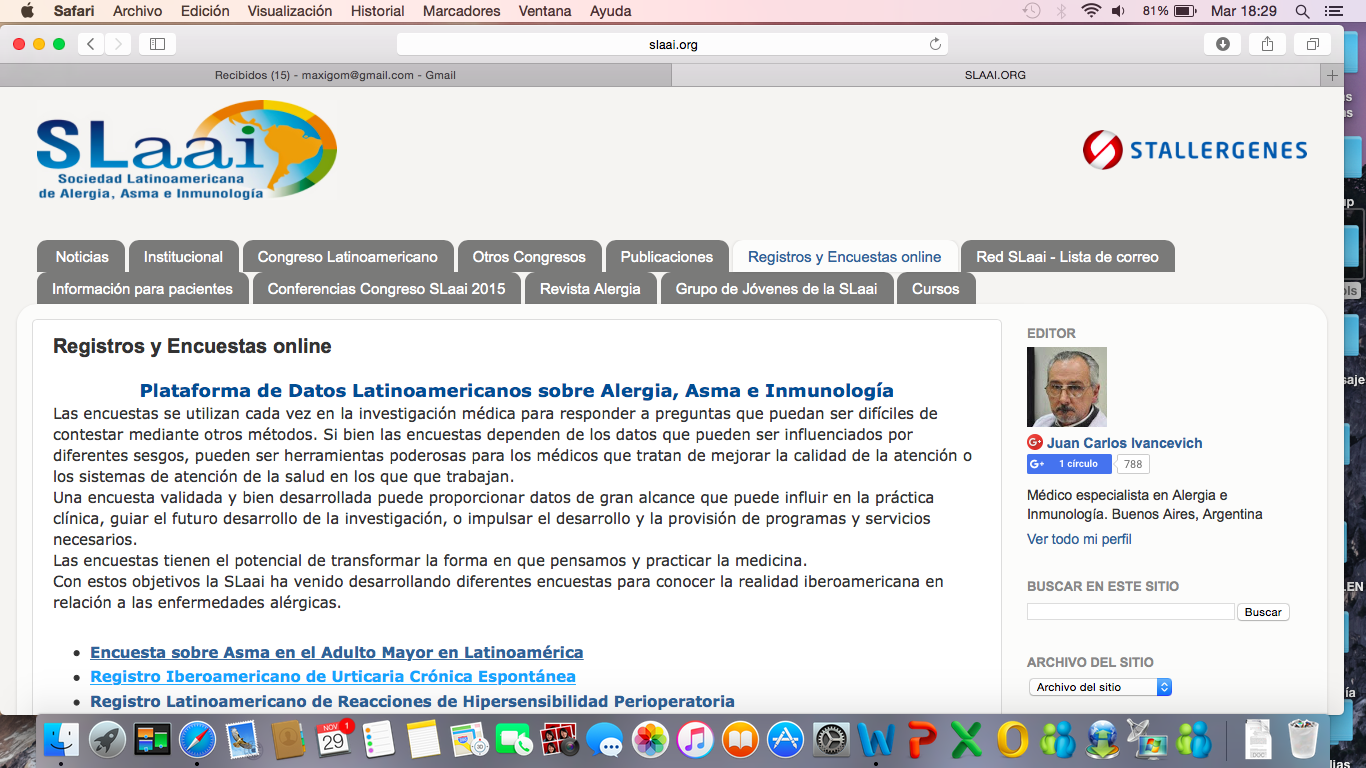


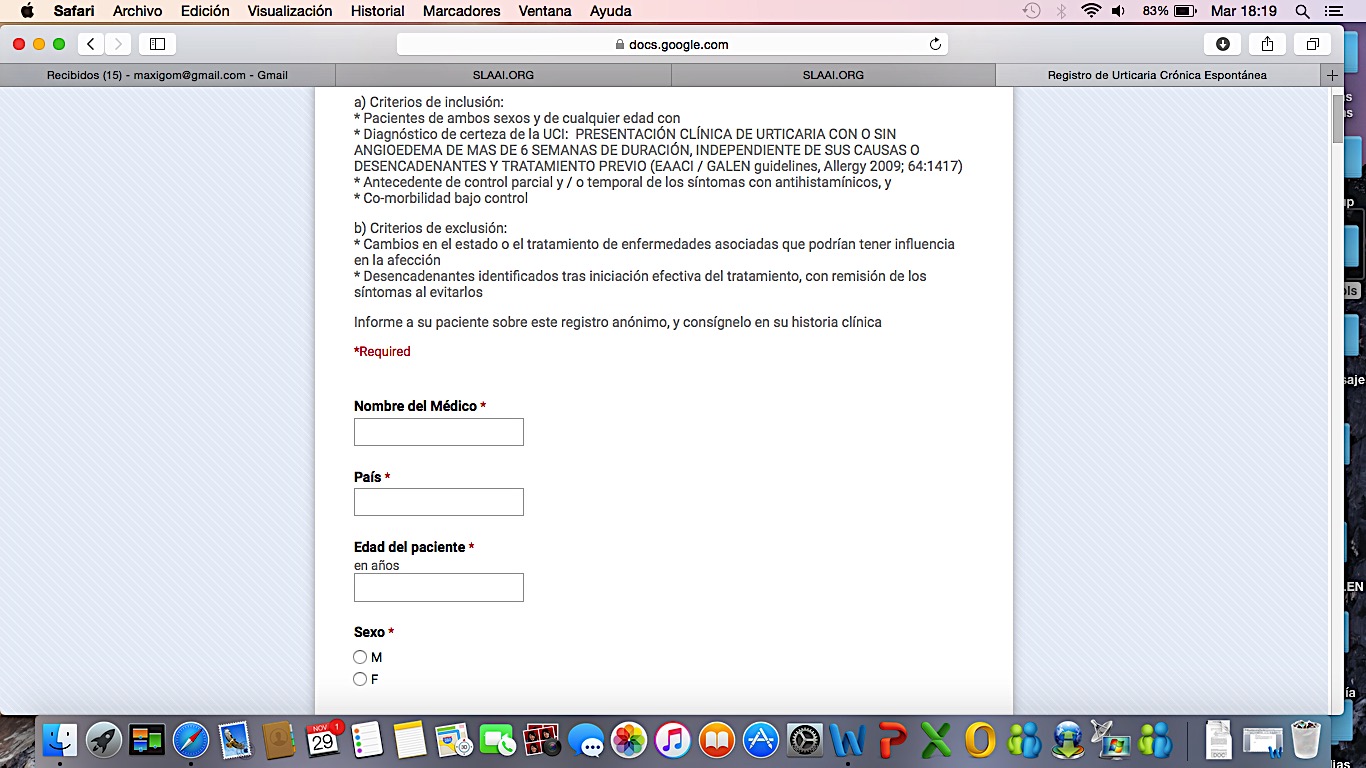


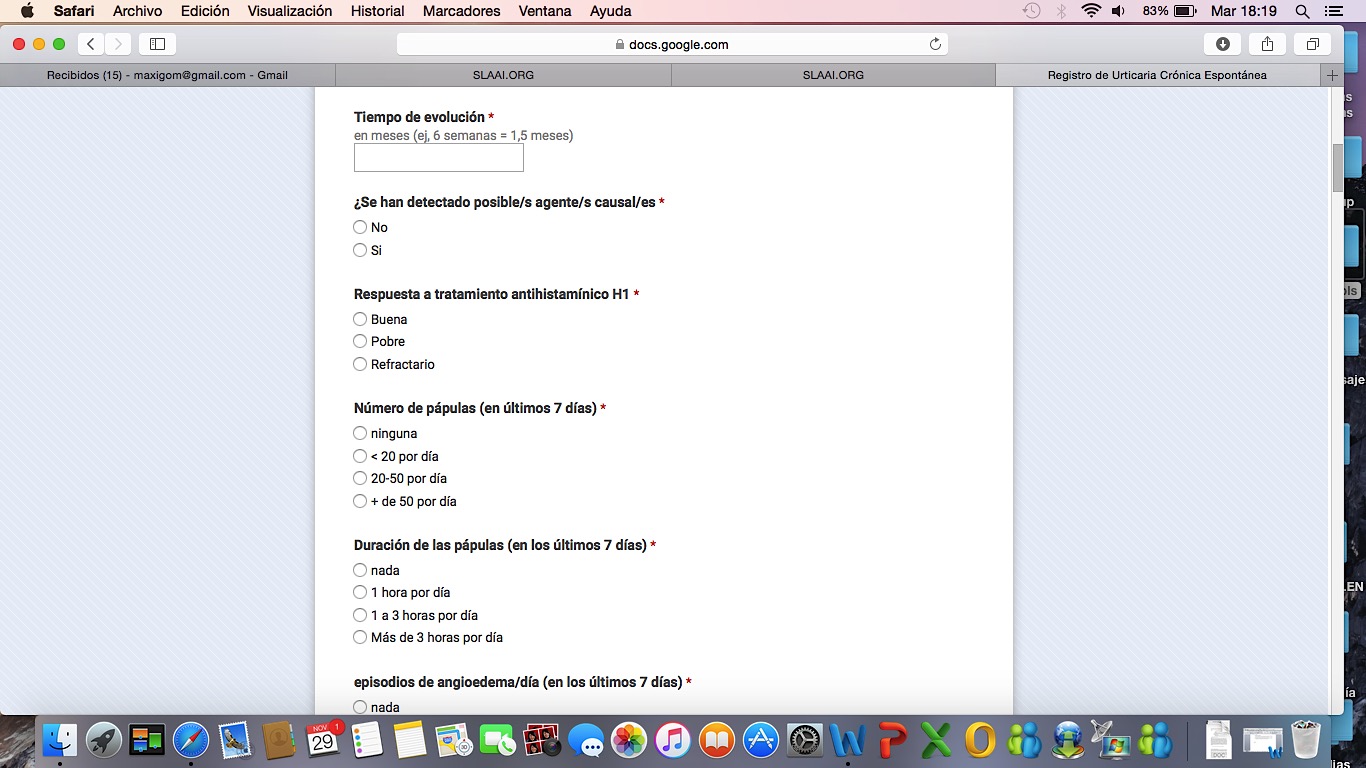


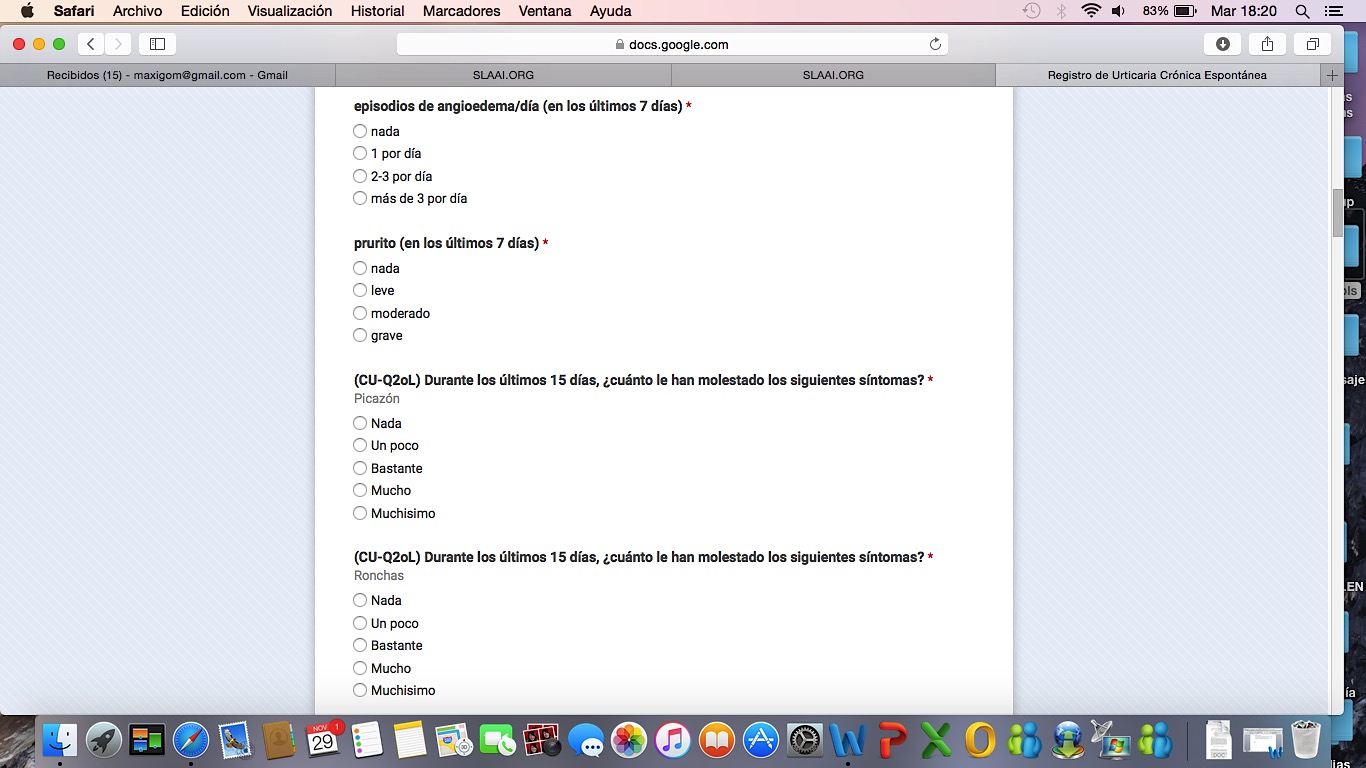


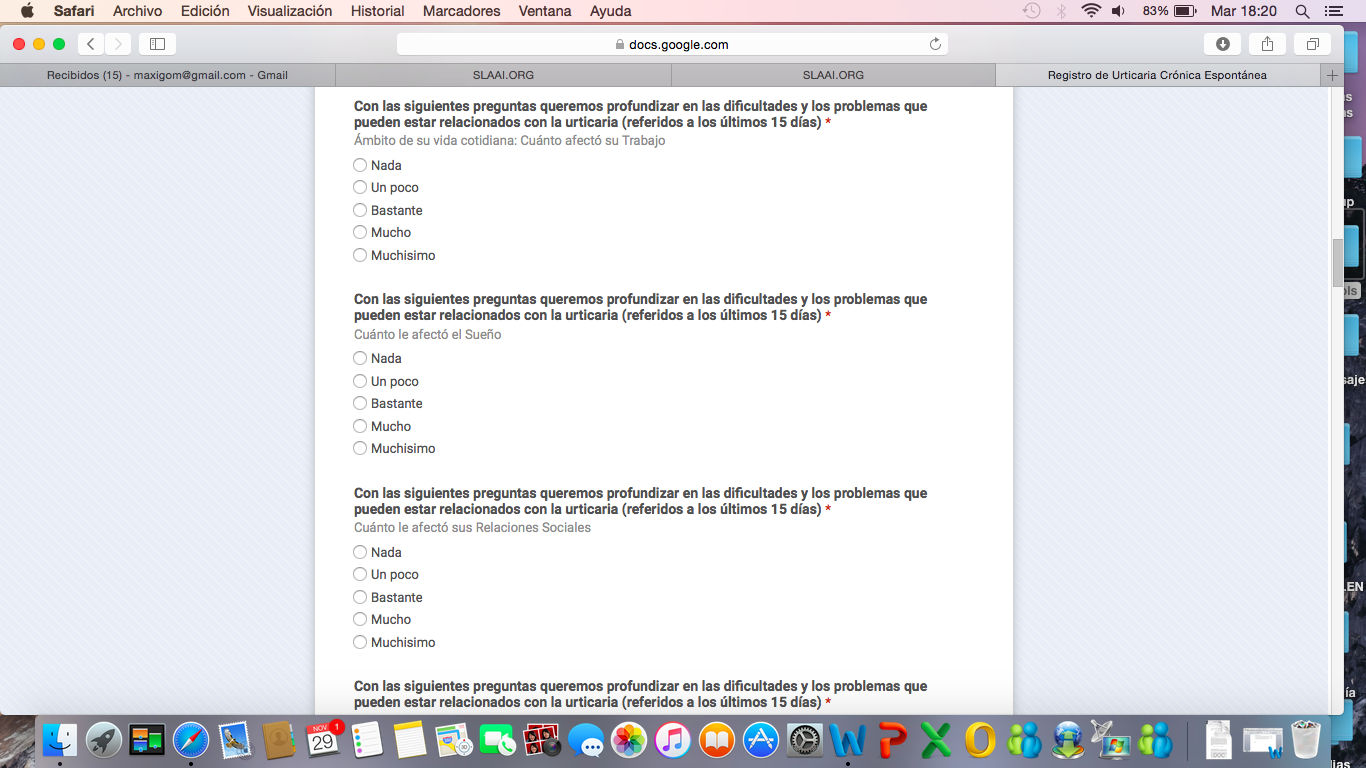


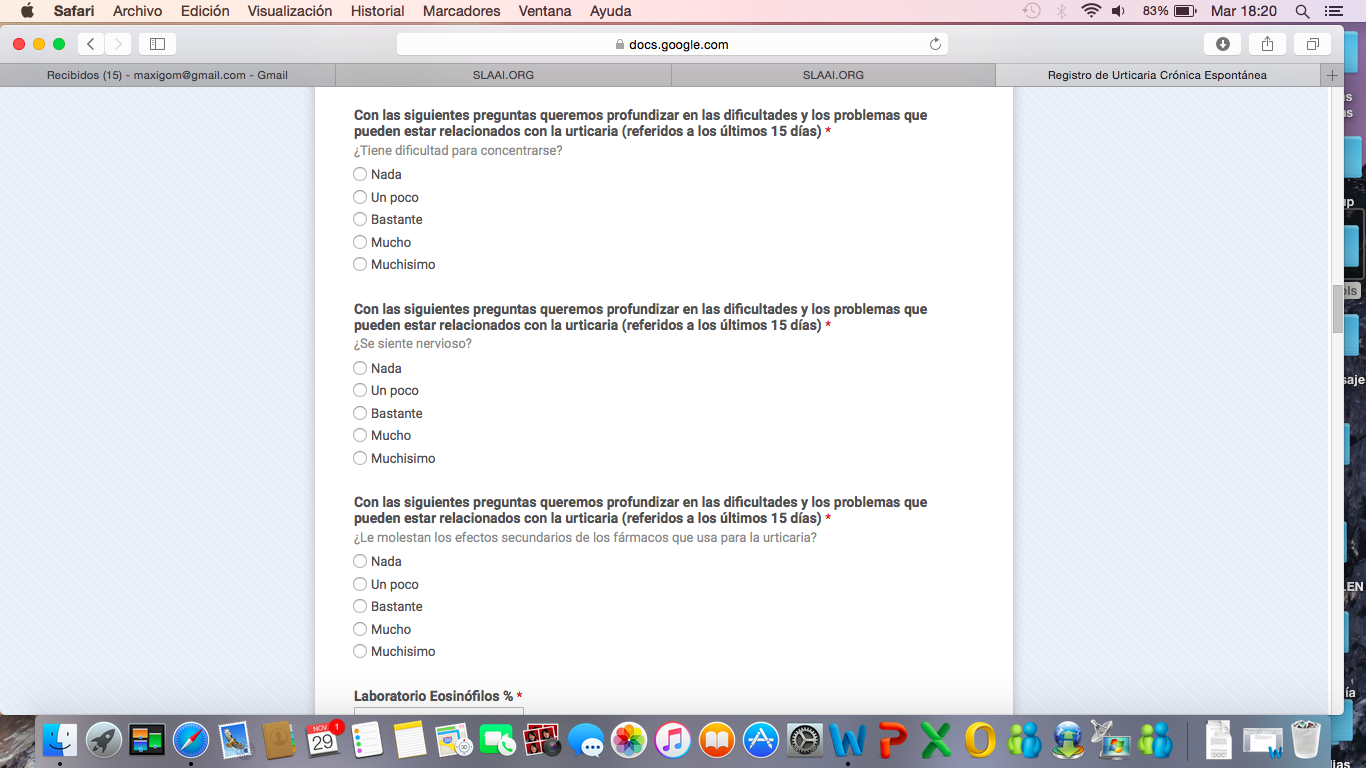


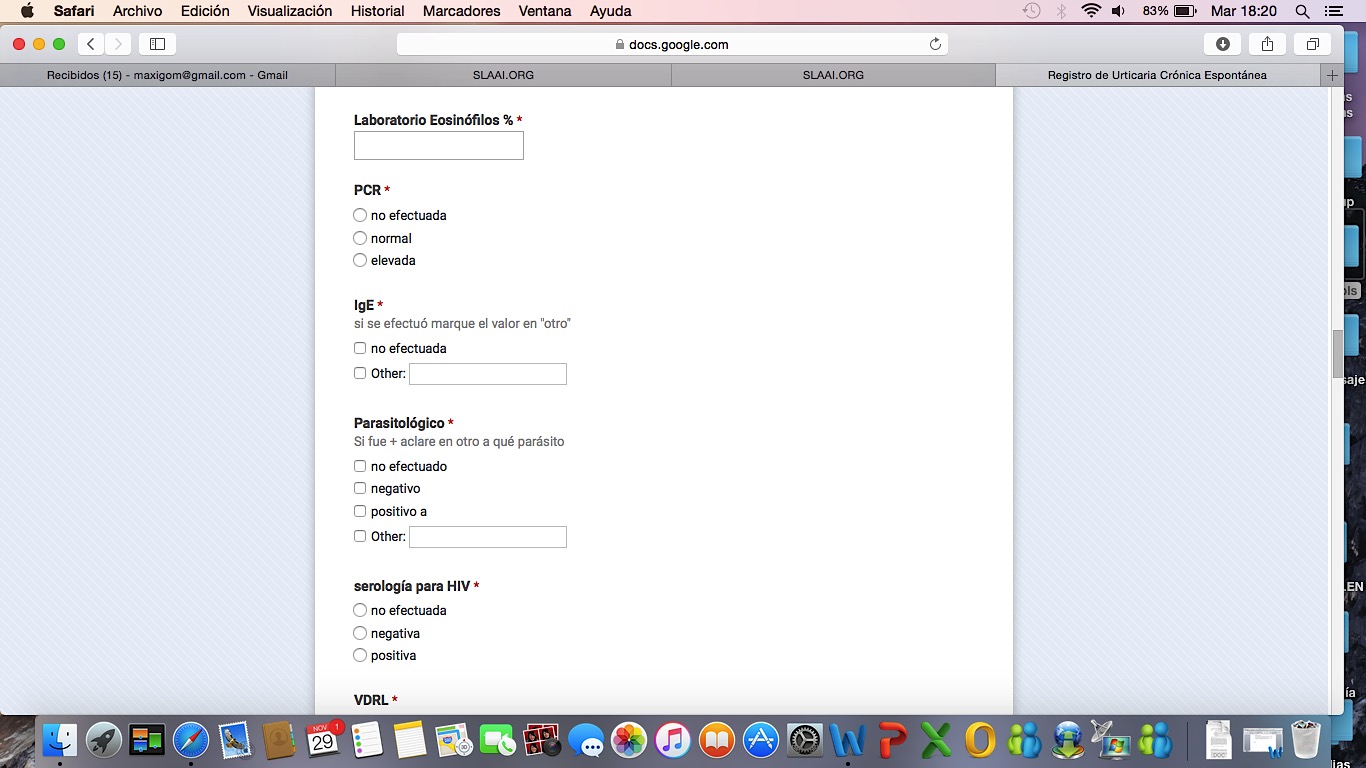


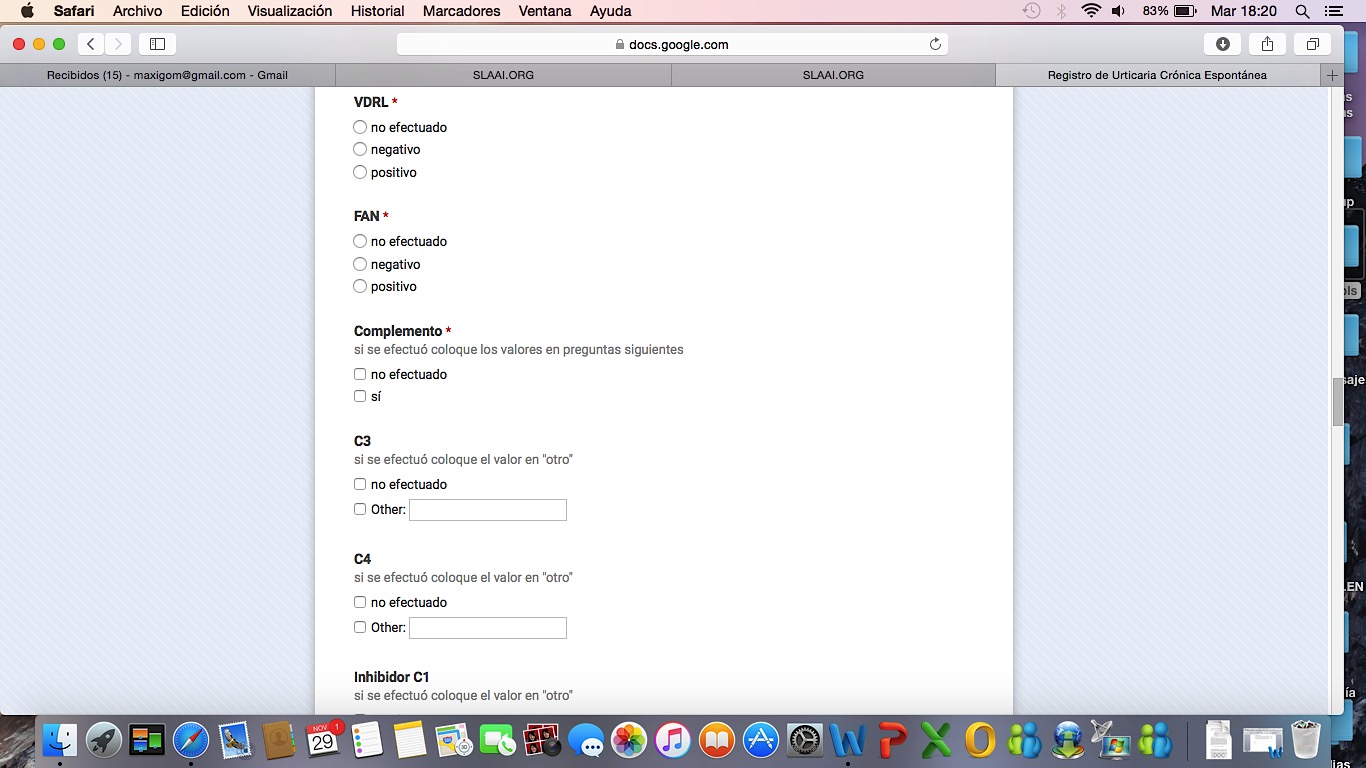


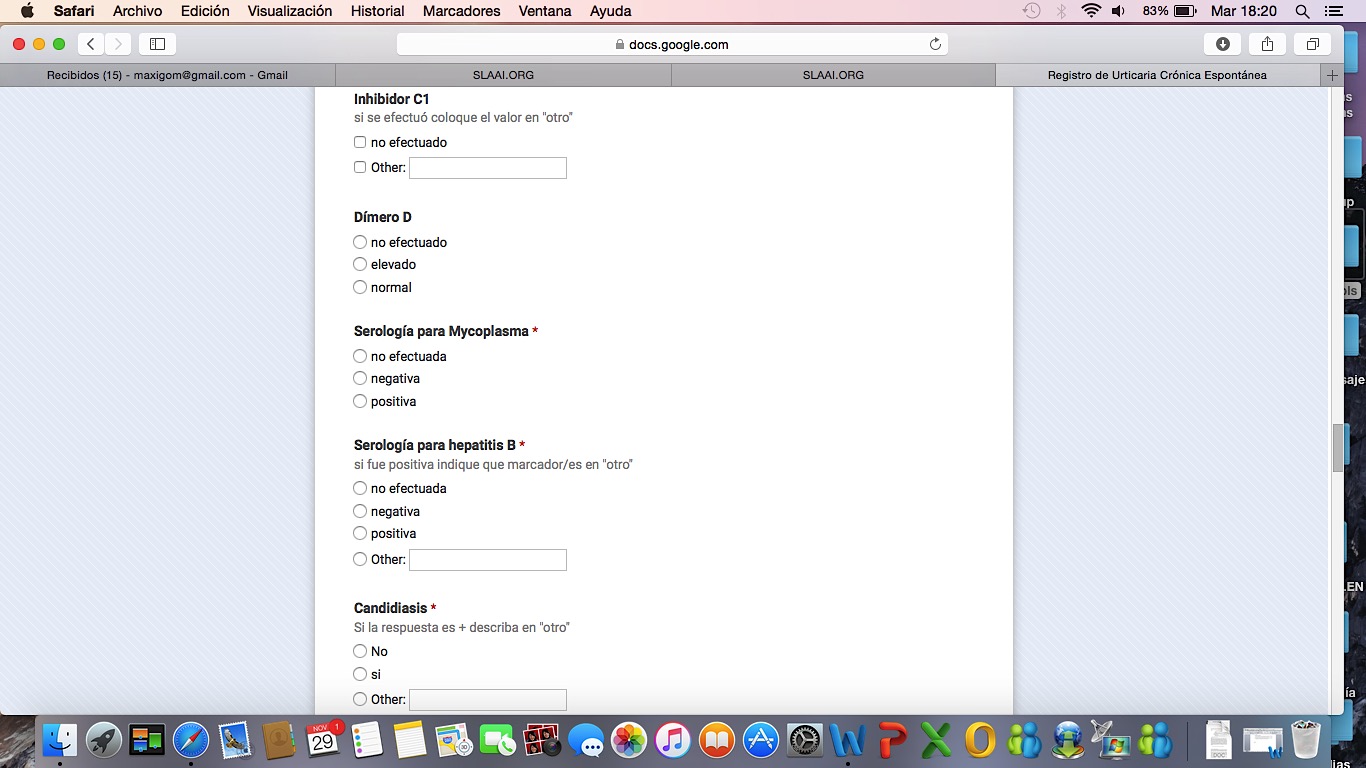


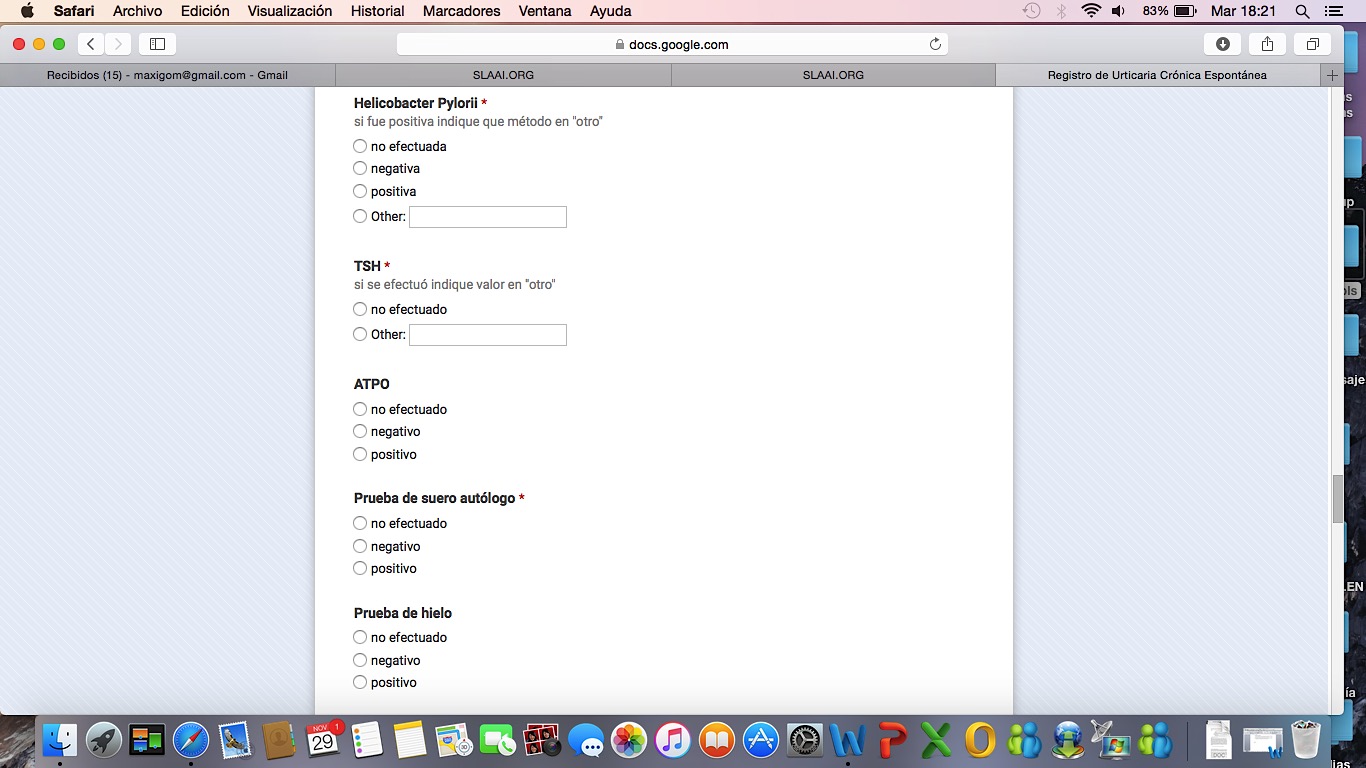


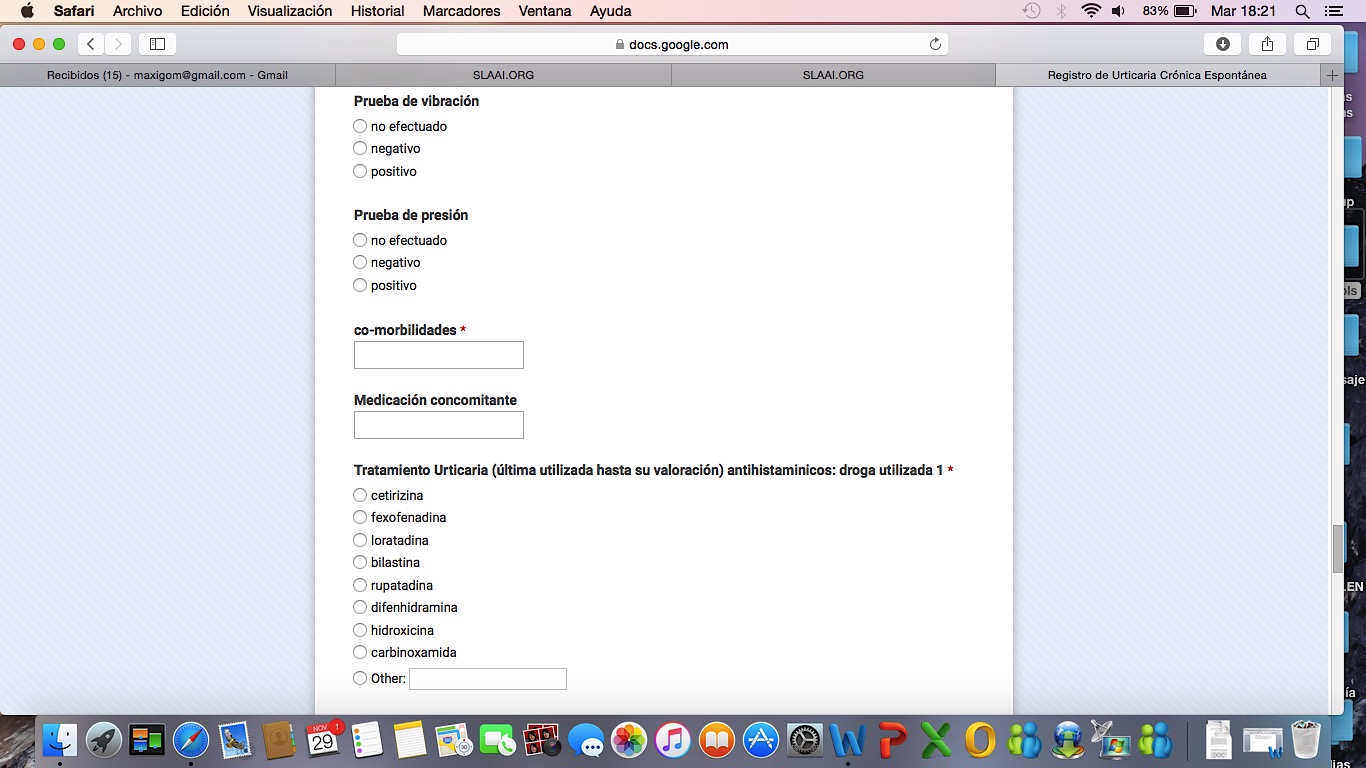


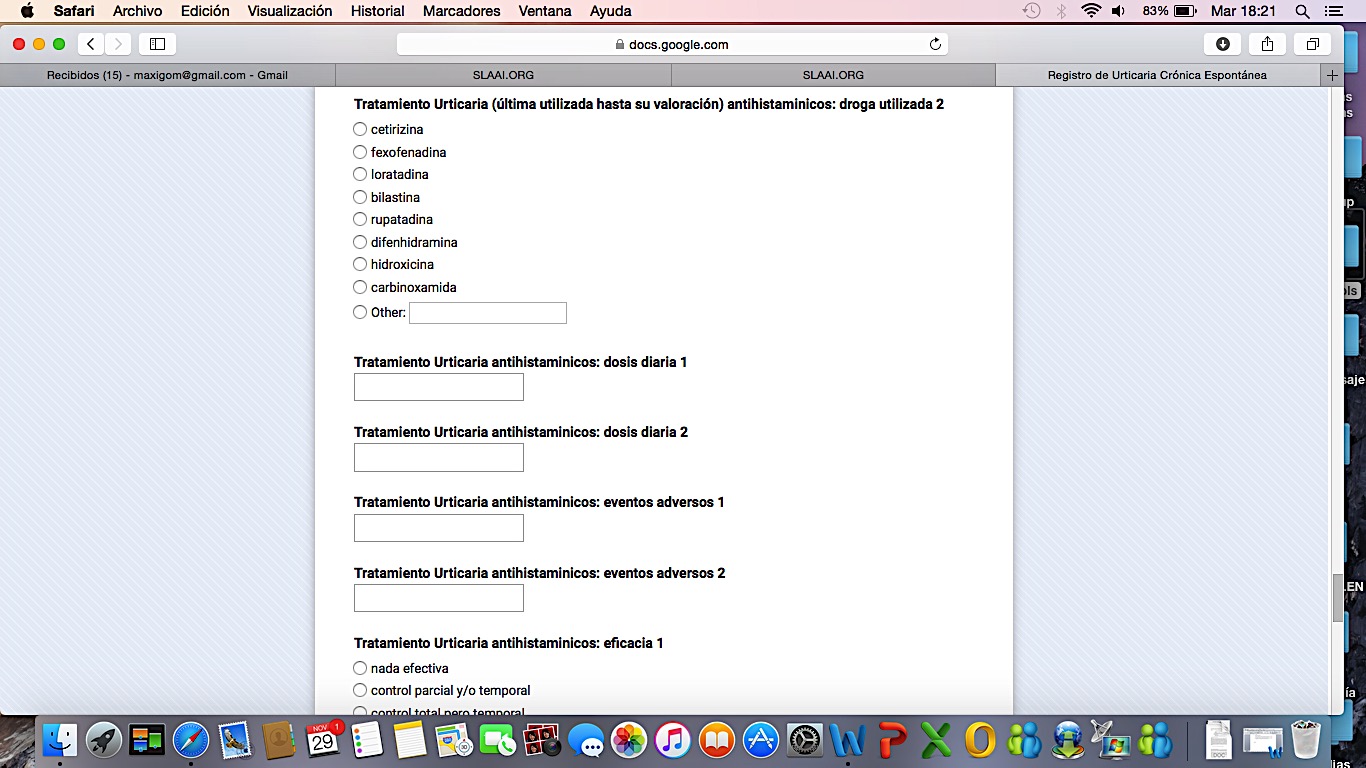


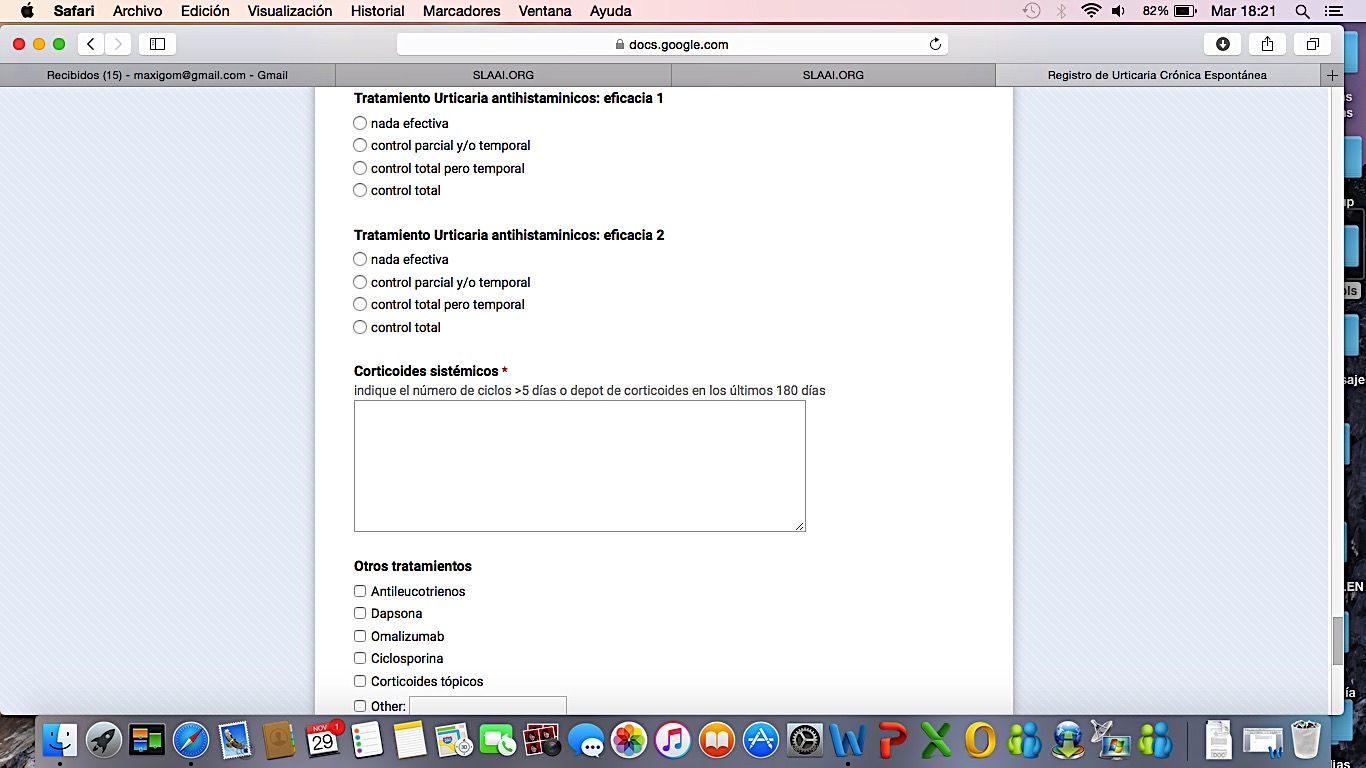


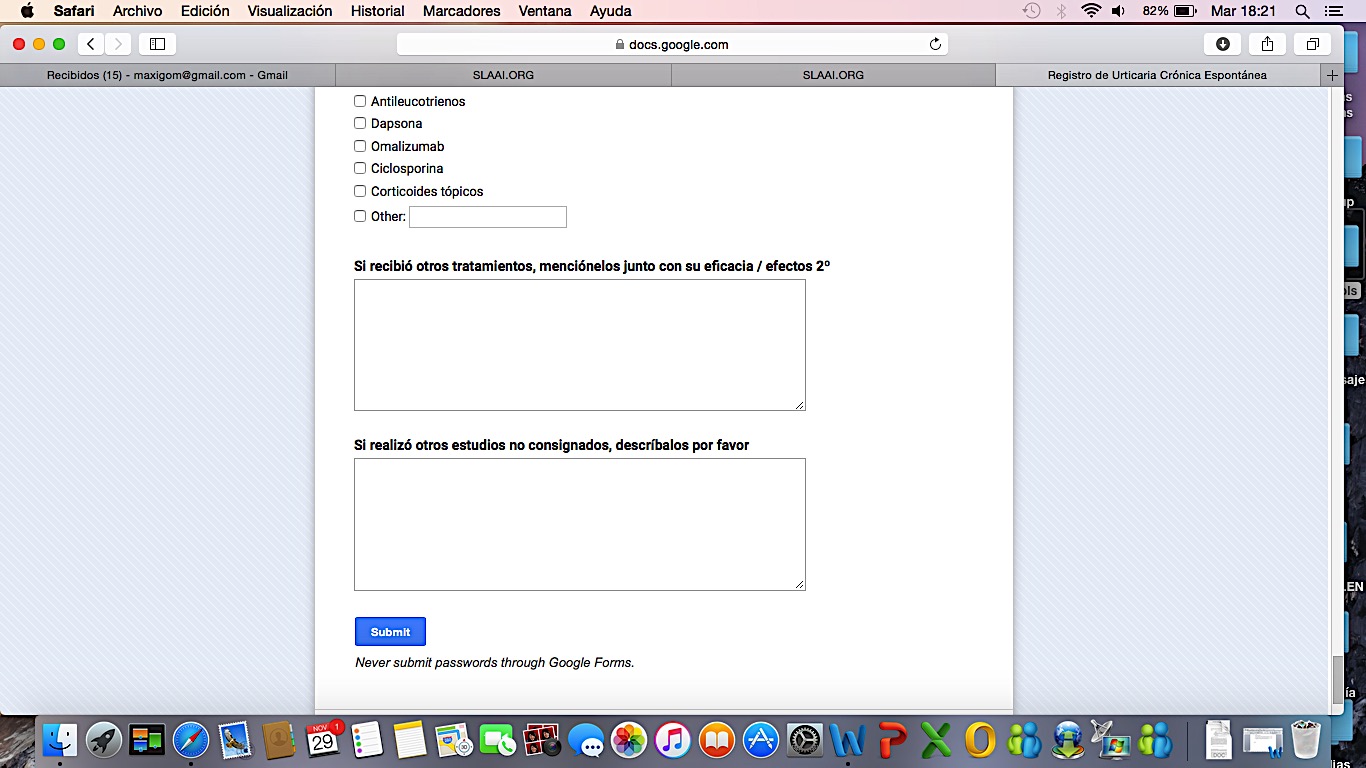

Supplement: Additional file 1: — The full content of the registry accessed on line. (DOCX 5526 kb) [file 40413_2017_147_MOESM1_ESM.docx]
